# Phenotypic Plasticity Strategy of *Aeluropus lagopoides* Grass in Response to Heterogenous Saline Habitats

**DOI:** 10.3390/biology12040553

**Published:** 2023-04-05

**Authors:** Abdulaziz M. Assaeed, Basharat A. Dar, Abdullah A. Al-Doss, Saud L. Al-Rowaily, Jahangir A. Malik, Ahmed M. Abd-ElGawad

**Affiliations:** Plant Production Department, College of Food and Agriculture Sciences, King Saud University, Riyadh 11451, Saudi Arabia

**Keywords:** functional traits, saline flat regions, halophytes, biomass allocation, desalination

## Abstract

**Simple Summary:**

Plants adapt themselves to harsh environmental conditions by changing morphological parameters through phenotypic plasticity. Plants modify their functional traits and allocate biomass to either tolerate or resist the stress caused by their variable habitats. In this study, we observed that *Aeluropus lagopoides,* being among the few halophytic palatable species of salt marshes, adapt to the harsh salt marshes of different eco-regions by significantly modifying its morphological and physiological traits. Due to this structural modification, this plant showed great potential to rehabilitate different inland and coastal saline flat areas (sabkha), taking saline agriculture and soil remediation into consideration.

**Abstract:**

Understanding the response variation of morphological parameters and biomass allocation of plants in heterogeneous saline environments is helpful in evaluating the internal correlation between plant phenotypic plasticity mechanism and biomass allocation. The plasticity of plants alters the interaction among individuals and their environment and consequently affects the population dynamics and aspects of community and ecosystem functioning. The current study aimed to assess the plasticity of *Aeluropus lagopoides* traits with variation in saline habitats. Understanding the habitat stress tolerance strategy of *A. lagopoides* is of great significance since it is one of the highly palatable forage grass in the summer period. Five different saline flat regions (coastal and inland) within Saudi Arabia were targeted, and the soil, as well as the morphological and physiological traits of *A. lagopoides,* were assessed. Comprehensive correlation analyses were performed to correlate the traits with soil, region, or among each other. The soil analysis revealed significant variation among the five studied regions for all measured parameters, as well as among the soil layers showing the highest values in the upper layer and decreased with the depth. Significant differences were determined for all tested parameters of the morphological and reproductive traits as well as for the biomass allocation of *A. lagopoides*, except for the leaf thickness. In the highly saline region, Qaseem, *A. lagopoides* showed stunted aerial growth, high root/shoot ratio, improved root development, and high biomass allocation. In contrast, the populations growing in the low saline region (Jizan) showed the opposite trend. Under the more stressful condition, like in Qaseem and Salwa, *A. lagopoides* produce low spikes in biomass and seeds per plant, compared to the lowest saline habitats, such as Jouf. There was no significant difference in physiological parameters except stomatal conductance (*g_s_*), which is highest in the Jizan region. In conclusion, the population of *A. lagopoides* is tolerant of harsh environments through phenotypic plasticity. This could be a candidate species to rehabilitate the saline habitats, considering saline agriculture and saline soil remediation.

## 1. Introduction

Different eco-regions with different climatic conditions can alter the available resources and conditions crucial to plant performance. The response of the plants to these environmental changes is through induced phenotypic changes [1]. Plant species with wide distribution among different eco-regions show large intraspecific variations in most functional and phenotypic traits [2,3]. The spatial variation in functional traits and their phenotypic plasticity can help plants persist under global climate change [4,5]. Variations in biotic and abiotic factors in different geographical regions can lead to morphologically and functionally different ecotypes [6]. Plants have to adjust to environmental heterogeneity through the plasticity of adaptive traits and respond to changes in light availability [7,8,9], water availability [9], nutrients [9,10], salinity [11,12], and temperature to survive and sustain in the soil-plant atmospheric continuum environment [13].

One major dependency for plant species to maintain their populations under variable environmental conditions is phenotypic plasticity [14,15]. Plants persist through this potential mechanism of phenotypic plasticity when faced with faster environmental changes and lead it toward homeostasis levels, thus allowing their proper functioning [16]. In the face of global warming, phenotypic plasticity has become a benchmark for understanding its potential for population persistence and adaptation [17]. Alterations in environmental conditions like light regimes, soil properties, humidity, and rainfall may shift several phenotypic traits [18,19,20]. Different plant populations exhibit adaptive plasticity in response to spatial variability of environmental conditions, such as climate and edaphic factors [21,22,23,24,25,26]. In heterogeneous environments, both abiotic and biotic factors can influence plant life-history traits (seed germination, growth, flowering, and reproduction) and adaptation (plasticity or differentiation) [15,27]. Measuring these phenotypic trait variations (both reproductive success and vegetative growth) and investigating the environmental variability of sites in which the population occurs is necessary to assess the adaptability and conservation status of the target species [28,29]. The plasticity of plants has a crucial impact on the community structure and dynamics, where plasticity alters many interactions between organisms and their abiotic and biotic factors of the environments [30]. The plant species characterized by phenotypic plasticity can colonize a wide range of habitats and modify the community structure as they tolerate different environmental factors.

Plants respond to variable environments by adjusting their multiple aspects of allocation and architecture, morphology, and physiology [1,31] to mitigate stress levels and increase the uptake of the limiting resources [32]. Biomass allocation among plant parts is driven by environmental conditions, which define many plant growth processes [33,34] and is related to the phenotypic characteristics of plants. Therefore, plant phenotypic plasticity can be used as a potential covariate for understanding biomass allocation [35,36].

In arid and semi-arid regions, water loss due to evapotranspiration increases the salt concentration in soil components [37], leading to more severe salinization issues. Natural saline habitats vary in salinity levels both spatially and temporally due to topography, soil properties, and micro-climate differences [38]. One such saline habitat is sabkha i.e.., a flat area of clay, silt, or sand with an overlying crust of soil [39]. The salt stress, moisture content, physio-chemical soil characteristics, and other environmental factors in saline areas tend to show relative stability with time [40], which has an extensive influence on community structure, plant morphological structure, and biomass allocation [41]. The biomass allocation of plants represents their growth and metabolism and affects the plant’s functional attributes [42].

Most salt marsh plant species are halophytes with a high degree of phenotypic variations, occupying a broad range of environmental conditions and possessing various traits to adapt to saline conditions [43,44,45]. Halophytic species have developed different mechanisms for regulating growth and development to ensure their survival in highly-saline inland or coastal areas, salt marshes, dunes, and desert habitats [46,47]. The distribution of some halophytic species is best correlated along a gradient of soil variables, such as salinity, moisture content, soil texture, organic matter, and calcium carbonate [48]. Halophytic grasses can tolerate salinity at a species-specific level and vary with the ecotype, region’s habitat, and specific environmental factors [14,49]. These halophytic species show adaptive phenotypic plasticity, enabling them to cope with different saline environments [50], as most traits exhibited considerable plasticity in response to different salinity stresses [51].

*Aeluropus lagopoides* (L.) Thwaites (Poaceae) is a stoloniferous halophytic perennial C_4_ photosynthetic grass ranging in distribution from Northern Africa (Morocco to Somalia), Italy, and Cyprus, through the deserts of the Middle East to Central Asia, Pakistan, and India [52]. In Saudi Arabia, it is found in different regions of saline coastal environments and inland areas. *A. lagopoides* was recorded from the inland wadi (valley) of Qareenah, Riyadh, saltmarsh areas of Qaseem and Jouf, and coastal zones of Salwa and Jizan region [53,54,55,56]. *A. lagopoides* is of economic importance as it is a palatable summer forage in arid areas as well as a sand stabilizer [14] and can be used for landscaping the urban areas of desert regions [57]. The plant withstands high salinity stresses up to 25 dS·m^−1^ and can adapt to heterogeneous environments due to structural adaptations and phenotypic trait modifications [58]. There is a considerable variation in environmental conditions of *A. lagopoides* habitats between different coastal and inland regions of Saudi Arabia, with variable effects on water relations, salinity, light, ambient temperature, and edaphic factors [55]. Consequently, the only dependency to maintain its populations under stressful environmental conditions is adaptive plasticity [14,15]. The ability of *A. lagopoides* to grow in different regions provides an excellent opportunity to study its phenotypic trait variations with respect to the regions in which it grows. However, the relationship between biomass allocation and root/shoot morphological strategies of *A. lagopoides* growing in different saline regions is unclear. Therefore, in this study, we aimed to explore the linkage of the variation in the functional traits of vegetative and root parts of *A. lagopoides* (i.e., phenotypic plasticity) with the differences in the habitats (edaphic factors). We aimed to clarify the following questions (1) how do morphological parameters of *A. lagopoides* synergistically change in response to habitat conditions? (2) what biomass allocation strategies did *A. lagopoides* have under different saline regions?

## 2. Materials and Methods

### 2.1. Surveyed Regions and Soil Analysis

**Figure 1:**
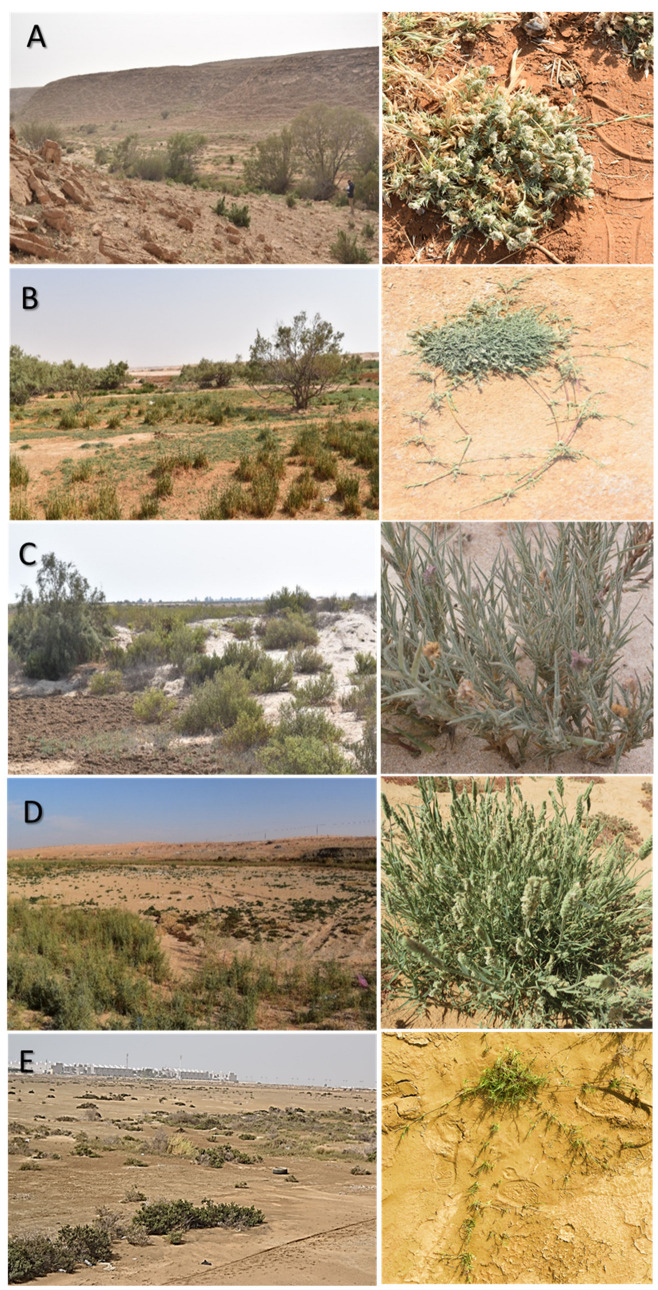
Different studied flat saline regions, (**A**) Qareenah, (**B**) Qaseem, (**C**) Salwa, (**D**) Jouf, and (**E**) Jizan. The left is an overview, and the right is a close view of *A. lagopoides*.

The populations of *A. lagopoides* were studied along Saudi Arabia during the years 2020–2021 and were found in five saline regions (Figure S1) were identified as follows: (1) Salwa (lowland coastal saline flat area), (2) Jizan (southern coastal saline flat area), (3) Qareenah (inland saline flat areas of wadi Hargan, Riyadh region), (4) Qaseem (an inland saline flat area of Al-Aushazia) and (5) Jouf ( an inland saline flat area in Domat Aljandal). The regions’ details are presented in Table S1. Each region was geographically different from the others as the shortest point-to-point distance between them was more than 300 km (Table S1 and Figure S2). Within each region, five distinct *A. lagopoides* patches were randomly selected for soil sampling and plant morphological traits measurements. From September to March (when *A. lagopoides* become fully flourished), three random plots (5 × 5 m) were selected within each patch (Figure 1). To assess the relationship between morphological and biomass allocation of the plant and resource allocation of the rhizosphere soil properties, three core soil sampling was selected. At three random points, three core soil samples were collected from three soil layers (0–15 cm, 15–30 cm, and 30–45 cm) within each plot. Each corresponding soil layer of these three soil samples was merged as one composite sample. A total of three composite soil samples represented each plot, and subsequently, a total of 9 samples from each patch. Hence, a total of 225 composite soil samples from all the studied region (5 regions × 5 patches × 3 plots × 3 layers) were collected. For soil moisture content, part of each sample was collected in duly labeled moisture tin, and the moisture content was immediately determined by the weight-loss method for all the samples.

For further analysis, all the soil samples were collected in plastic bags, duly labeled, and transferred to Range Science Lab., College of Food and Agriculture Sciences, King Saud University, Riyadh, Saudi Arabia. All the soil samples were spread over separate plastic sheets, air-dried at room temperature, and sieved through a 2 mm sieve to remove any debris, and the soil samples were analyzed similarly to the previously reported approach of Dar et al. [55]. In brief, the soil texture was determined using the hydrometer method [59]. Soil organic matter was determined by wet combustion with dichromate at 450 °C [60]. Soil water extracts (1:5) were prepared for the estimation of soil electrical conductivity (EC) and pH [60]. Soluble inions (Cl^−^ and SO_4_^2−^) were determined by the titration method, while soluble cations (Ca^2+^, Mg^2+^, Na^+^, and K^+^) were determined using a flame photometer according to Rhoades [61].

### 2.2. Morphological Traits Measurements

Within each plot, five fully matured individuals were randomly selected for morphological parameters, including both on-field and off-field functional trait measurements were recorded. A total of 375 individuals (5 regions × 5 patches × 3 plots × 5 individuals) were targeted for the measurements. In the field, shoot length, number of tiller/plant, number of leaves/tiller, number of spikes/plant, spike length, top internode length of the main tiller, number of stolon/plant, and top internode stolon length were measured.

On the other hand, after taking the field measurement, the same individuals were excavated and collected in labeled plastic bags. The bags were brought in an ice-cool box to the Range Science Laboratory, College of Food and Agriculture Sciences, King Saud University, Riyadh, Saudi Arabia, for other measurements like leaf area, biomass, and root morphological parameters. Plant samples were separated into root and shoot systems. The leaf area of five leaves of each individual was measured using the WinDIAS system (Delta-T Devices Ltd., Cambridge, UK). Also, the average area and biomass of five spikes of each individual were measured. The root system of all the individuals was thoroughly washed, and their main root length, root hair length, total root area, and root dry matter were measured. Based on these measurements, specific leaf area (SLA) was determined as the ratio of leaf area to leaf dry mass. Leaf dry matter content (LDMC) was calculated as the ratio of leaf dry mass to saturated fresh mass [62]. Leaf thickness was calculated as the ratio (SLA × LDMC^−1^). For resource allocation, root/shoot ratio, root mass fraction, and shoot mass fraction were calculated. These functional traits were selected to assess the response and plasticity of *A. lagopoides* to the environmental factors within different regions, according to Perez-Harguindeguy et al. [63].

### 2.3. Determination of Ecophysiological Traits

Before the targeted plant individuals were excavated, chlorophyll fluorescence, chlorophyll content, leaf temperature, and stomatal conductance were measured. Chlorophyll fluorescence was measured with an Opti-Sciences OS30p+ chlorophyll fluorometer (Opti-Sciences, Hudson, NY, USA). Fluorescence was measured at midday (11.00–13.00 h, solar time). Chlorophyll fluorescence, initial fluorescence (F0), maximum fluorescence (Fm), and variable fluorescence (Fv) were determined, and the ratios of Fv/F0 and Fv/Fm were calculated using MINI-PAM fluorometer (Heinz Walz GmbH, Effeltrich, Germany). The minimum and maximum dark-adapted fluorescence (F0, Fm) and Fv/Fm (where Fv = Fm—F0) were obtained after the leaves of the plants were dark-adapted for at least 20–25 min [64].

In-situ stomatal conductance (gs) was measured using a steady-state diffusion porometer (model SC-1, Decagon Devices, Inc., Pullman). Each day before measurements, the porometer was calibrated, and gs was measured on the adaxial surface of a fully developed penultimate leaf in the afternoon (13:00–15:00 h). The chlorophyll content was measured according to the method of Lichtenthaler and Wellburn [65] with some modifications. About 0.5 g of the fresh plant sample was extracted by acetone, and the content of chlorophyll a (Chl. a), chlorophyll b (Chl. b), and total chlorophyll were determined by measurements of the absorbance at 663 and 645 nm with the UV-VIS spectrophotometer (SHIMADZU, Kyoto, Japan, UV1800).

### 2.4. Statistical Analysis

To compare the various traits of *A. lagopoides* and determine the significant variations among regions, the data for the plant functional traits and ecophysiological parameters were analyzed by one-way ANOVA with the region as a factor. However, the soil properties were analyzed for three-way ANOVA with the regions, soil layers, and samples as factors. The ANOVA was performed using Statistix 8.1 software. The soil samples were also used as a factor in the soil analysis to check the homogeneity of soil samples within the studied region. Mean values were compared by the Duncan Multiple Range (DMR) test using SAS 9.1.3. The standard error (SE) was calculated for each mean value.

To correlate the various plant traits (vegetative and reproductive) with each region, a data matrix of the shoot, root, and reproductive traits from the five studied regions was subjected to principal component analysis (PCA). The plant morphological traits were plotted as loading vectors in a bi-plot, while the region was plotted as observations.

On the other side, in order to assess the relationship between the soil parameters of each region and morphological traits, two datasets were prepared; one of the various morphological traits and the second of the soil variables of the studied regions at the three layers (0–15 cm, 15–30 cm, and 30–45 cm). These two datasets were subjected to ordination analysis using canonical correspondence analysis (CCA). Also, the agglomerative hierarchical clustering (AHC) and heatmap were performed based on the data of the top layer soil parameters and the morphological traits of *A. lagopoides* populations within the five studied regions. The AHC was performed based on the Pearson correlation coefficient and weighted pair-group average agglomeration method. PCA and AHC were performed using the XLSTAT software program (version 2018, Addinsoft, NY, USA), while CCA was produced using the MVSP software program, ver. 3.1 [66].

## 3. Results

### 3.1. Soil Layer Variations among the Regions of A. lagopoides

Soil analysis revealed significant variation (*p* < 0.05) among the five studied regions supporting the growth of *A. lagopoides* for all measured parameters, except for HCO_3_ as well as among the soil layers (Table 1). The Qaseem, Salwa, and Qareenah regions had the highest and comparable pH values for the top layer soil (0–15 cm), while the Jizan and Jouf regions attained the lowest values of the pH (Table 1). In general, the pH values decreased significantly (*p* = 0.0219) with the soil depth in all studied regions.

The soil salinity is highly significantly varied among soil layers for all regions (*p* = <0.001). The soil of the Qaseem region generally had the highest values of EC (25.95 dS/m for the top layer, 10.28 dS/m for the middle layer, and 6.32 dS/m for the lower layer), cation (Ca^2+^, Mg^2+^, and Na^+^) and anions (Cl^−^) of all the regions as well as for all the soil layers. However, the values of K^+^ and SO_4_^2−^ varied in significance from layer to layer among the locations, the highest value of K^+^ for the top layer (20.22 meq/L) being in the Qaseem region and the below two layers (9.95 meq/L for the top layer and 3.34 meq/L for the lower layer in Jouf region (Table 1). The highest value of SO_4_^2−^ for the top layer (71.8 meq/L) was recorded in the Jizan region. However, Qaseem attained the highest amount of Cl^−^ (237.60, 73.30, and 51.00 meq/L for the top middle and lower layers, respectively) for all three layers compared to other locations. The cations and anions of the Jouf region soil showed a trend of lower concentration with soil depth. The soil of the Qareenah region attained the highest content of CaCO_3_ among all regions, and the content increased with the increase in soil depth. On the other hand, the Qaseem region attained the highest organic matter content for the top layer (1.78%), followed by the Qareenah region for all the soil layers (1.63%, 0.98%, and 0.91% for the top, middle, and lower layers, respectively).

Regarding soil texture, the sand content is highest in Salwa for all three layers and the lowest in silt content, while the Qaseem region had the lowest values of sand and the highest of silt for all three soil layers. Clay content was highest in Qaseem for the top layer (16.80%), while it was highest in the Jizan region for the layer of 15–30 cm (13.58%) and the layer of 30–45 cm (12.32%). Moisture content showed a significant difference (*p* < 0.0001) among layers, and it was highest in the Qaseem region for all three layers compared to other regions’ respective layers. Overall, the results of soil analyses showed that the highest soil characteristic values trend from top to bottom layer (0–15 > 15–30 > 30–45) for all the regions, with some minor exceptions.

### 3.2. Morphological Traits Variations among the Studied Regions of A. lagopoides

By comparing the five studied regions of *A. lagopoides*, significant differences were determined for all tested parameters of the morphological and reproductive traits as well as for the biomass allocation, except for the leaf thickness, where no significant difference was observed (Table 2).

#### 3.2.1. Shoot Traits

**Figure 2:**
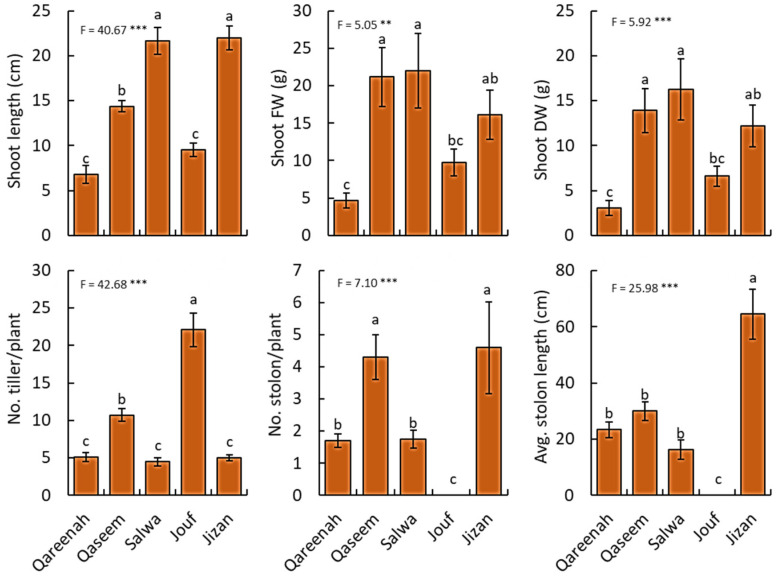
Comparison of shoot traits of *Aeluropus lagopoides* growing in different saline flat regions of Saudi Arabia. Values are average (*n = 75*), and the bar represents the standard error. Different letters among regions showed significant differences at *p* < 0.05 after Duncan’s test. ** *p* < 0.01*** *p* < 0.001.

A highly significant difference in the shoot length and biomass was observed among regions (*p* < 0.001). Moreover, the shoot length of *A. lagopoides* growing in the Jizan and Salwa regions was the highest, while it was lowest in Qareenah and Jouf regions (Figure 2). For shoot biomass (fresh and dry weight), Qassem and Salwa regions attained the highest values, while the lowest values of shoot fresh and dry weight were assessed in the Qareenah region (4.68 g and 3.05 g, respectively). The number of tillers per plant, stolon number per plant, and stolon length showed highly significant differences (*p* < 0.001) among regions of *A. lagopoides.* The number of tillers per plant was higher in the Jouf region, while the number and length of stolon were higher in Jizan and Qaseem regions (Figure 2). Jouf region attained the lowest values of stolon measurements.

#### 3.2.2. Root Traits

**Figure 3:**
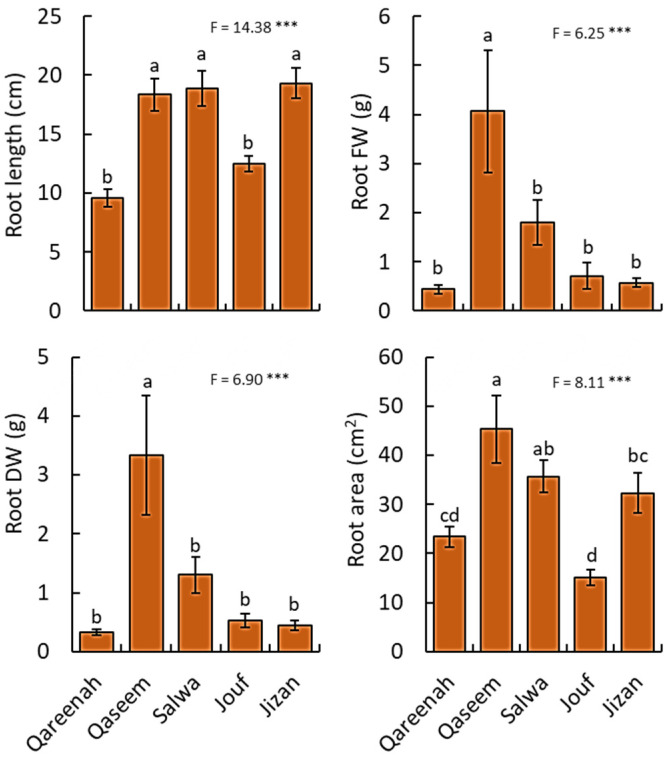
Measured root traits for *Aeluropus lagopoides* growing in different saline flat regions of Saudi Arabia. Values are average (*n = 75*), and the bar represents the standard error. Different letters among regions showed significant differences at *p* < 0.05 after Duncan’s test. *** *p* < 0.001. FW: fresh weight, DW: dry weight.

All studied root traits of the samples showed highly significant differences (*p* < 0.001) among the five studied regions (Figure 3). For root length, Qaseem, Salwa, and Jizan regions attained the highest values, while Qareenah and Jouf had the lowest root length. For biomass, *A. lagopoides* growing in the Qaseem region attained the highest root fresh and dry weight. Moreover, the highest value of the root area was determined for the *A. lagopoides* growing in the Qaseem region (45.27 cm^2^), followed by Salwa (35.71 cm^2^) and Jizan (32.31 cm^2^) regions (Figure 3).

#### 3.2.3. Leaf Traits

**Figure 4:**
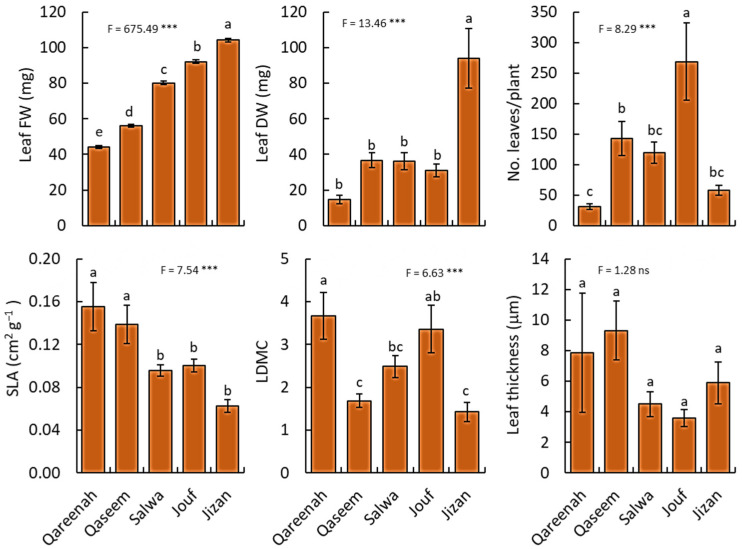
Comparison of leaf traits for *Aeluropus lagopoides* growing in different saline flat regions of Saudi Arabia. Values are average (*n = 10*), and the bar represents standard error. Different letters among regions showed significant differences at *p* < 0.05 after Duncan’s test. SLA: specific leaf area, LDMC: leaf dry matter content. FW: fresh weight, DW: dry weight. *** *p* < 0.001, and “ns” for *p* > 0.05.

All leaf traits of *A. lagopoides* plants showed highly significant variation among regions (*p* < 0.001), except for leaf thickness which did not vary significantly (*p* = 0.29) from one region to another (Figure 4).

The Jizan region attained the highest values of leaf biomass (fresh and dry weight), while the Qareenah region had the lowest values of both leaf fresh and dry weight. The number of leaves per plant was highest for the population of the Jouf region, while the Qareenah region attained the lowest number of leaves. In contrast, the Qareenah region attained the highest values of specific leaf area (0.16 cm^−2^ g^−1^), and the Jizan region showed the highest value of leaf dry matter content (90.16%). However, no significant difference in leaf thickness was found in all studied regions.

#### 3.2.4. Reproductive Traits

**Figure 5:**
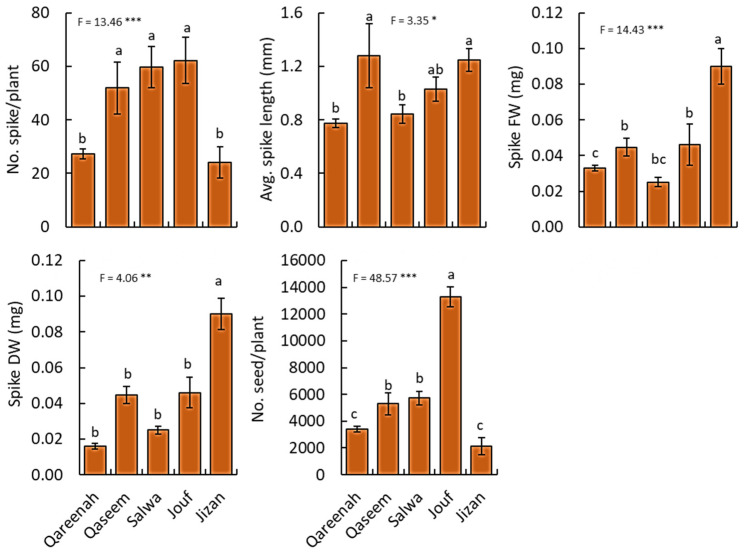
Reproductive traits for *Aeluropus lagopoides* growing in different saline flat regions of Saudi Arabia. Values are average (*n = 75*), and the bar represents the standard error. Different letters among regions showed significant differences at *p* < 0.05 after Duncan’s test. * *p* < 0.05, ** *p* < 0.01, *** *p* < 0.001. FW: fresh weight, DW: dry weight.

All the measured reproductive traits of *A. lagopoides* showed a highly significant difference (*p* < 0.001) among the studied regions (Figure 5). Regarding spike numbers, the populations of the Jouf region showed the maximum production of spikes and seeds per plant, while Qareenah and Jizan attained the lowest values (Figure 5). The average spike length of the Qaseem regions was the highest, while Qareenah and Salwa regions attained the lowest values of the spike length. For spike biomass (fresh and dry weight), the Jizan region attained the highest values (0.09 and 0.06 mm, respectively), while Qareenah and Salwa regions showed the lowest biomass among the studied regions.

### 3.3. Variation in Ecophysiological Parameters of A. lagopoides among Different Regions

**Figure 6:**
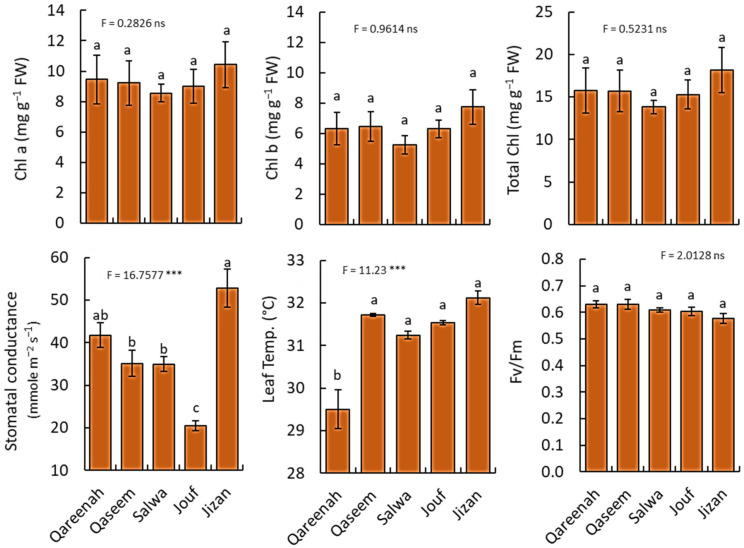
Ecophysiological parameters of *Aeluropus lagopoides* growing in different saline flat regions of Saudi Arabia. Values are average (*n = 75*), and the bar represents the standard error. Different letters among regions showed significant differences at *p* < 0.05 after Duncan’s test. *** *p* < 0.001, and “ns” for *p* > 0.05. FW: fresh weight.

The photosynthetic pigments (chlorophyll *a,* chlorophyll *b*, total chlorophyll) varied slightly among different regions but had no significance (Figure 6).

Similarly, there is no significant difference in Fv/Fm, and all values were under 0.79. On the other hand, stomatal conductance showed a highly significant difference among the regions (*p* < 0.001), as the Jizan region had the highest stomatal conductance (52.88 mmole m^−2^ s^−1^), while the Jouf region had the lowest value (20.46 mmole m^−2^ s^−1^). Moreover, a highly significant difference was observed among the studied regions for the *A. lagopoides* leaf temperature.

### 3.4. Biomass Allocation of A. lagopoides in Response to Different Habitats

**Figure 7:**
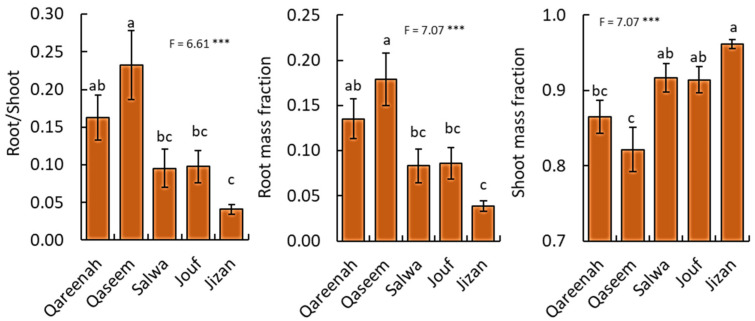
Biomass allocation among *Aeluropus lagopoides* collected from different saline flat regions of Saudi Arabia, based on dry matter. Values are average (*n = 75*), and the bar represents the standard error. Different letters among regions showed significant differences at *p* < 0.05 after Duncan’s test. *** *p* < 0.05.

The biomass proportion of plant parts in *A. lagopoides* was significantly different (*p* < 0.001) among the studied regions (Figure 7). Concerning root:shoot ratio, the population of the Qaseem region showed the highest value (0.23), followed by Qareenah (0.16), while the population of the Jizan region attained the lowest values of root/shoot ratio (Figure 7). Similarly, the root mass fraction showed the same pattern, where *A. lagopoides* of the Qaseem region showed the highest root mass fraction (0.18), followed by Qareenah (0.13), while *A. lagopoides* of the Jizan region attained the lowest value (0.04). In contrast, the population of the Jizan region showed the highest shoot mass fraction (0.96), while the population of the Qaseem and Qareenah regions attained the lowest values.

### 3.5. Correlation Analysis among Functional Plant Traits, Regions, and Soil Variables

#### 3.5.1. Plant Functional Traits-Regions Correlations

**Figure 8:**
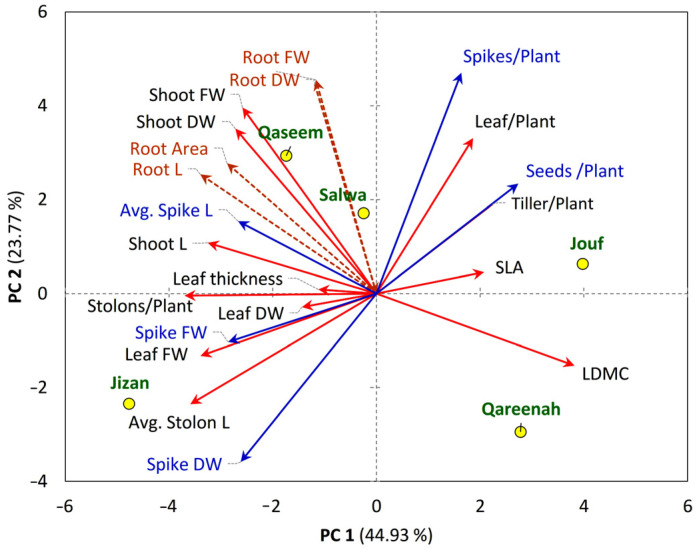
Principal component analysis (PCA) of the measured traits (shoot, represented with red arrows, root represented with brown arrows, reproductive traits represented with blue arrows of *Aeluropus lagopoides* within different saline flat regions (represented with yellow circle) of Saudi Arabia.

The principal component analysis (PCA) revealed the existence of a close correlation between different morphological traits of *A. lagopoides* and the studied regions (Figure 8). The PCA revealed that the number of spikes and number of seeds per plant correlate with the number of tillers and leaves per plant (Figure 8). The *A. lagopoides* growing in Qaseem and Salwa regions are closely correlated and showed a positive correlation with root and shoot biomass. The root traits (root biomass, root area, and root length) are separated on the upper-left side of the PCA biplot, where it showed correlations to each other as well as with shoot biomass. Spike length showed a correlation with the shoot length. The *A. lagopoides* population of the Jizan region showed a substantial correlation with the leaf biomass, spike biomass, and stolon length, where spike biomass revealed a correlation with the leaf biomass as well as the stolon length (Figure 8). The Jouf region showed a significant positive correlation with specific leaf area, number of leaves per plant, number of tillers per plant, and number of seeds per plant (Figure 8). However, leaf dry matter content was the only morphological trait closely correlated to the Qareenah region.

#### 3.5.2. Correlations among Soil Variables, Plant Functional Traits, and Regions

**Figure 9:**
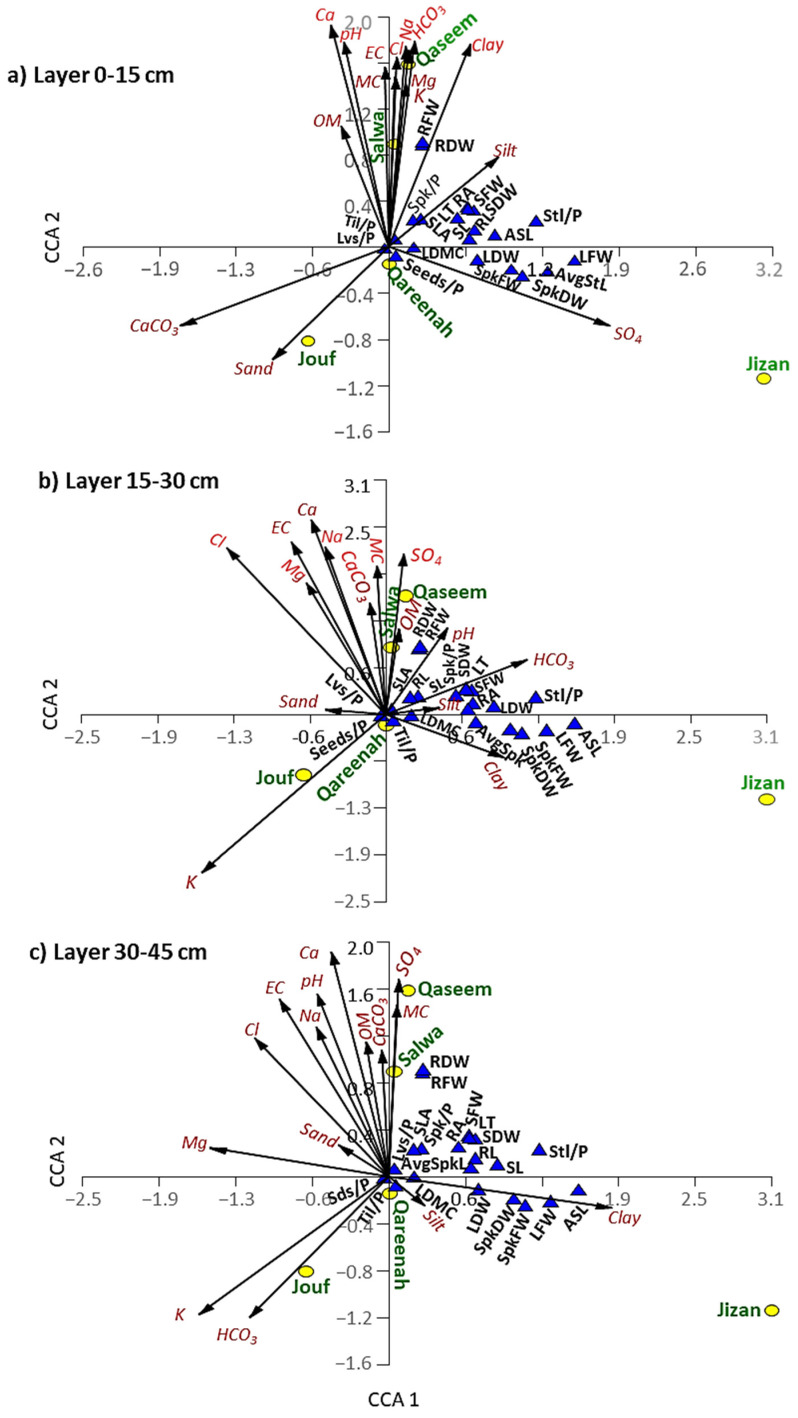
Canonical correspondence analysis (CCA) showing the correlations among the soil variables of different layers separately ((**a**): 0–15 cm, (**b**): 15–30 cm, and (**c**): 30–45 cm layers), regions, and morphological traits of *A. lagopoides*. SFW: shoot fresh weight, SDW: shoot dry weight, RFW: root fresh weight, RDW: root dry weight, lvs/*p*; number of leaves per plant, SLA: specific leaf area, Spk/*p*: number of spikes per plant, AvgSpkL: average spike length, RA: root area, LT: leaf thickness, SL: shoot length, RL: root length, Stl/*p*: number of stolon per plant, LDMC: leaf dry matter content, LFW: leaf fresh weight, LFW: leaf fresh weight, LDW: leaf dry weight, SpkFW: spike fresh weight, SpkDW: spike dry weight, ASL: average stolon length.

The data of soil variables of each layer of each region and the functional traits were correlated using CCA (Figure 9). In general, Qassem and Salwa regions show a close correlation to each other, while Jizan is segregated alone on the lower-right side of the CCA biplot. Jouf region was different from other regions for the soil profile of the three layers (upper, middle, and lower) and separated on the lower-left side of the CCA biplot. Finally, the Qareenah region was separated at the center of the CCA biplot, revealing no specific correlation to any parameters.

Regarding the top layer of the soil (0–15 cm), the Qassem and Salwa regions showed a close correlation to most of the soil parameters that are correlated together, such as moisture content, pH, salinity, organic matter, Na, Cl, Mg, K, and Ca. (Figure 9a). Jizan region showed a close correlation to sulfate content, where it showed a correlation to leaf and spike biomass traits. The soil of the top layer in the Jouf region is different and separated on the lower-left side of the CCA biplot, where it showed a correlation to calcium carbonate and sand contents, and it showed a negative correlation with all morphological traits (Figure 9a).

The heatmap correlation analysis, based on the soil data of the top layer, revealed that root biomass (root fresh weight and root dry weight) has a significant correlation with Ca^2+^, Na^+^, Cl^−^, Clay, and moisture content, while root area showed a significant correlation to only clay content (Figure S3). On the other hand, specific leaf area showed a significant positive correlation with organic matter, while leaf dry matter content showed a correlation to calcium carbonates.

The leaf thickness revealed a significant positive correlation to Na and K contents. The spike biomass showed a significant correlation with sulfate content. However, leaf dry weight showed a significant negative correlation with K, bicarbonates, and organic matter (Figure S3).

For the middle layer of the soil (15–30 cm), the Qaseem and Salwa regions again showed a close correlation with moisture content, salinity, organic matter, Na, and Ca, while the Jizan region showed a correlation to the clay content. However, the Jouf region showed a close correlation to potassium ions but a negative correlation with all studied plant traits (Figure 9b). Pearson’s correlation heatmap of the middle layer showed that root biomass significantly correlates with moisture content and sulfate (Figure S3). Moreover, the specific leaf area showed a significant positive correlation with organic matter like the top layer. The number of tillers and seeds per plant showed a significant correlation with the potassium ion. However, leaf biomass revealed a significant negative correlation with organic matter and calcium carbonates (Figure S3).

The PCA analysis of the lower layer of the soil (30–45 cm) revealed a different pattern compared to the upper and middle layers (Figure 9c). In this soil layer, the plant traits did not show a positive correlation to any soil parameters, except for clay, which correlated to the leaf biomass, spike biomass, and average stolon length of the *A. lagopoides* growing in Jizan (Figure 9c). Pearson’s correlation heatmap of the lower layer showed a similar pattern to the middle layer (Figure S3).

### 3.6. Cluster Analysis of Regions Based on Soil and Plant Functional Traits

**Figure 10:**
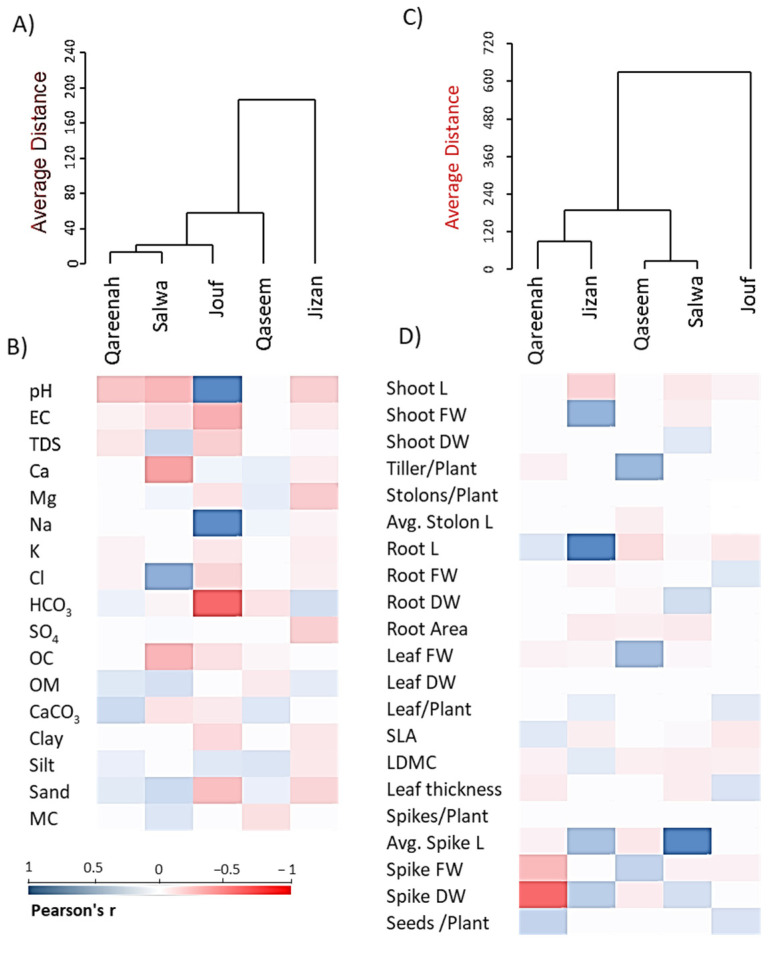
Agglomerative hierarchical clustering (AHC) and heatmaps of the studied parameters within different saline flat regions of *Aeluropus lagopoides*. (**A**) AHC and (**B**) heatmap based on the soil variables, (**C**) AHC and (**D**) heatmap based on the morphological and reproductive traits. EC: electrical conductivity, OM: organic matter, SLA: specific leaf area, LDMC: leaf dry matter content.

The hierarchical clustering for soil variables showed that Qareenah and Salwa regions are quite similar and showed a little pit correlation to the Jouf region (Figure 10A). However, the Jizan region differs in its soil characteristics from other regions. Regarding the plant functional traits, the Qareenah and Jizan regions are closely related, while Qaseem and Salwa regions showed a close correlation to each other (Figure 10C). However, the Jouf region is unique in the functional traits of *A. lagopoides.*

The combination of clustering with heatmap analysis revealed that the *A. lagopoides* populations growing in the Jouf Region showed a negative correlation with all the soil variables except Na^+1^ and pH (Figure 10B), while the Salwa region showed a positive correlation with organic matter and chloride. On the other hand, the heatmap analysis of morphological traits revealed that *A. lagopoides* populations growing in the Qareenah region showed a negative correlation with spike dry weight and fresh weight. In contrast, the Jizan region was positively correlated with root length and fresh weight. The Salwa region revealed a positive correlation with spike length (Figure 10D).

## 4. Discussion

Desert vegetation faces various ecological constraints like high temperature, soil salinity, and low soil moisture due to low precipitation, making the desert region a challenging environment for plants to grow [67]. Under stressful environments, desert grasses show specific structural and functional modifications in morphological and physiological characteristics to thrive well in such harsh environments [68]. The present study revealed that the various saline flat areas inhabited by *A. lagopoides* differed significantly in soil physicochemical characteristics from region to region as well as with soil depth (up to 30 cm), i.e., among layers (Table 1). These edaphic factors shaped these study sites’ community structure and species association [55]. Salt stress can cause a reduction in water potential in soil and can induce osmotic stress in plants [69]. The structural and functional mechanisms of differently adapted populations of a desert halophyte (*A. lagopoides*) were studied for its survival and growth in hyper-arid-saline environments. When species undergo specific drought events and variable edaphic factors like soil salinity and high pH, they restrict their growth by utilizing energy for survival rather than further growth and development [70,71]. The continuity of environmental effects and the distance between the studied regions may support the likelihood for specific characteristics to become fixed in this grass over time.

Soil physical and chemical parameters of the habitats of all these five studied regions were significantly different, indicating the adaptive potential of *A. lagopoides* to cope with variable environmental conditions. Thus *A. lagopoides* plant faces the dual environmental stress of salt and water scarcity. Most of the soil physio-chemical characteristics values like salinity, pH, organic matter, cations, and some anions like Cl_,_ and HCO_3_, in all three soil layers of the inland saline flat area of the Qaseem region, were high, followed by the inland saline flat area of the Qareenah region (Table 1). The coastal Salwa and Jouf regions were moderately saline, and the least saline was the coastal saline flat area of the Jizan region (Table 1). The soil salinity, pH, cations, and anions were highest in the top soil layer and decreased significantly with depth, where this observation could be attributed to the high evaporation rate [72]. However, the cations and anions increase in value as soil depth increases in the Jouf region which could be ascribed to waterlogging at the site of the Jouf region [73].

Based on these soil characteristic variations, *A. lagopoides* evolved independently to these different salt levels among regions and responded quite differently relating to their morphological and physiological parameters (Figure 2, Figure 3, Figure 4, Figure 5 and Figure 6). In this study, the highly saline Qaseem region (25.95 dS·m^−1^) has the stunted growth of aerial parts of *A. lagopoides* (shoot length, stolon length, leaf biomass). This could be due to the high salinity level in the soil of this region. The same less shoot height was reported in *Aeluropuslittoralis* under different salinity [74]. This growth restriction of the aerial part of the plant is an essential morphological adaptation because a short-statured plant may conserve the energy required for vital metabolic processes [75]. In contrast, *A. lagopoides* growing in an inland saline flat area of the Qaseem region had improved root development, such as increased root length, high root fresh weight, root dry weight, and more root area than the low saline Jizan region. The investment in root development is an essential line of defense against salt stress [76] and determines the capacity of the plants to obtain water and nutrients [77,78]. Generally, root parameters increase under salinity in most halophytic species [79], while the opposite is true for glycophytic and less salt-tolerant species [80]. *A. lagopoides*, an indicator species of highly saline soils, grow well and uses Na^+^ for many physiological processes [81,82]. The well-developed root system of *A. lagopoides* in highly saline habitats may have provided additional benefits to this plant under physiological drought in extracting moisture from the deeper soil layer, a common phenomenon in plants subjected to limited water availability [83]. This observation is supported by data on biomass allocation, where the *A. lagopoides* growing in the Qaseem region attained the highest root/shoot ratio as well as the root mass fraction (Figure 7). This reflects that when *A. lagopoides* is subjected to more salinity, it invests more energy in root development compared to shoot.

On the other side, most aerial parts like shoot length, shoot fresh weight, and shoot dry weight of the Jouf region were stunted, and the soil was moderately less saline than in the Qaseem region (Figure 2). This may be due to the combined effect of salinity and drought (low soil moisture content). Previous studies also reported reduced stem elongation of *Abies alba* [84] under saline and drought-prone environments. The number of tillers in *A. lagopoides* in the Jouf region is significantly more than in other regions. This, again, may be due to the low soil salinity of the Jouf region. The high osmotic stress of the salt outside the roots reduces the formation rate of new leaves and tiller productions [85] in moderate to high saline regions. *A. lagopoides* tends to produce thick leaves with low fresh and dry weight and low LDMC in the Qaseem region (Figure 4). This may be due to the highest soil salt content and the strongest degree of salinization in this habitat. The leaves of *A. lagopoides* became fleshy and developed a lot of water storage palisade tissues and water transport tissues [86]. Firstly, a fleshy leaf structure can also dilute the cell salt ion concentration to avoid its toxic effect. Secondly, it can also increase vacuole concentration and decrease water potential via ion regionalization, thus alleviating the water stress caused by salt stress [87]. Therefore, forming thick leaves in highly saline habitats may be a survival strategy for inland salt marsh plants to adapt to the harsh environment for a longer time.

Regarding reproductive traits, *A. lagopoides* showed a highly significant difference among the studied regions. Under the more stressful condition, like in Qaseem and Salwa, *A. lagopoides* produce more spikes while showing low spikes in biomass and seeds per plant, compared to the lowest saline habitats, such as Jouf (Figure 5). This could be explained as the plant, under stressful conditions, invests more in seed production and does not make an effort to produce spikes, which is a strategy to maintain more seeds that is more important to the survival of the species in harsh environments [88]. The spike number and inflorescence biomass of *Spartina alterniflora* have been reported to be decreased with increasing salinity [89]. However, the Jouf region showed maximum production of spikes and seeds per plant, while Jizan, in spite of the lowest production, attained the highest value for fresh and dry weight. This could be due to high spike and seed size and mass which the plants tradeoff for spike number and seeds based on the resources available in the habitats they adapt. Plasticity in reproductive investment is also an important trait in varying environments because changes in spike length, seed number, weight, and size directly influence plant fitness [90].

Different levels of salinity adversely influence stomatal conductance (g_s_). *A. lagopoides* exhibited declined stomatal conductance (g_s_) with increasing salinity (Figure 6). It depicts that stomatal conductance is an effective strategy to prevent water loss for maintaining the normal function of photosynthetic activity under saline conditions. However, stomatal conductance decreased in the low saline and low moisture content Jouf region. The decrease in stomatal conductance could reduce water loss, which is an adaptation mechanism by plants in dry soil conditions [91]. The highest efficiency of the PSII photochemistry (*Fv/Fm*) method has been extensively used to detect plant stress differences in response to environmental challenges and, consequently, to screen tolerance levels to environmental stress [92]. The *Fv/Fm* of *A. lagopoides* did not show a significant difference among the studied regions, while all values were under 0.79 (Figure 6), meaning that plants are under stress conditions.

Under limited resources, *A. lagopoides* improves its fitness by balancing biomass allocation between aboveground and belowground plant parts and synergistic morphological variation between shoot and root systems. In variable environments, plants’ developmental traits and biomass allocation strategies are responses toward morphological characteristics of a plant’s location adaptation to resource heterogeneity [93]. Vegetative (especially leaves) and roots are essential for plants to acquire resources. Plant morphologically changes with the environmental gradient to obtain most of the resources and strategies ecologically to adapt to environmental changes [94]. Under both water and salt stress in the Qaseem region, the roots of *A. lagopoides* adopted a strategy of root development and expansion to obtain resources to improve their adaptive ability. Thus *A. lagopoides* formed a good root architecture and produced a well-developed network of fibrous roots by increasing the root area and increasing root biomass. Our results are in agreement with the conclusion that an increase in soil salt concentration increased root development [95].

Overall, the present results demonstrated that the morphological architecture and biomass allocation of *A. lagopoides* are significantly affected in different saline flat area regions based on habitat heterogeneity vis a vis moisture content and salinity as a strategy for adaptation to harsh environments.

## 5. Conclusions

The morphological, reproductive, and physiological traits of *A. lagopoides* in the present study show plasticity with the change in the environmental conditions of saline flat area habitats. The Regions with high salinity, such as Qaseem and Salwa, showed the highest values of most of the shoot and root traits. However, the population of *A. lagopoides* in Qaseem and Salwa showed more spikes and lower spikes in biomass and seeds per plant compared to the lowest saline habitats, such as Jouf. Under stressful conditions, i.e., high salinity, the grass produces more seeds instead of spike biomass or other morphological traits. This plasticity reflects the strategy of *A. lagopoides* to cope with the harsh/saline environment. The ability of *A. lagopoides* to change its morphology with the variations in the environmental conditions enables it to colonize, dominate, and shape the community structure within the salt marsh habitat of different regions. The data on biomass allocation in the present study revealed that *A. lagopoides* invests more energy toward roots than shoots under stressful conditions. Due to the extensive fibrous root network, this plant could be a promising candidate as a soil stabilizer in saline flat areas during the summer season. Based on our data, we can conclude that the population of *A. lagopoides* shows great potential to rehabilitate the saline habitats of inland and coastal saline flat regions, taking saline agriculture, saline soil remediation, and stabilization into consideration, particularly this grass flourished in the dry summer season when these habitats are devoid of forage vegetation. Further study is recommended to evaluate the transplantation of this promising forage grass on a large scale in saline rangeland habitats degraded due to heavy grazing of a few palatable halophytic species.

## Figures and Tables

**Table 1:** Physical and chemical properties of different soil layers supporting *Aeluropus lagopoides* in different regions.

Parameters	Layer	Region					*p*-Value
		Qareenah	Qaseem	Salwa	Jouf	Jizan	
pH	0–15 cm	8.38 ± 0.18 A,a	8.39 ± 0.141 A,a	8.39 ± 0.140 A,a	8.19 ± 0.108 B,C,a	8.09 ± 0.220 C,a	0.0011 **
	15–30 cm	8.47 ± 0.04 A,a	8.16 ± 0.027 B,b	8.22 ± 0.022 AB,b	8.02 ± 0.051 C,b	8.10 ± 0.192 C,a	
	30–45 cm	8.19 ± 0.07 B,b	8.15 ± 0.034 B,b	8.27 ± 0.115 A,B,a,b	8.02 ± 0.097 C,b	7.88 ± 0.207 C,b	
*p*-value	0.0219 *						
EC (dS·m^−1^)	0–15 cm	15.39 ± 0.92 B,a	25.95 ± 3.87 A,a	7.29 ± 0.17 C,a	2.74 ± 0.13 C,b	1.032 ± 0.07 D,a	<0.0001 ***
	15–30 cm	5.17 ± 0.78 B,b	10.28 ± 1.83 A,b	5.25 ± 0.26 B,b	4.52 ± 1.03 C,a	1.10 ± 0.16 D,a	
	30–45 cm	3.87 ± 0.46 B,c	6.32 ± 1.14 A,c	3.53 ± 0.58 C,c	3.59 ± 1.12 C,a	0.94 ± 0.07 D,b	
*p*-value	<0.0001 ***						
Ca^2+^ (meq/L)	0–15 cm	19.90 ± 2.69 C,a	39.86 ± 3.74 A,a	31.87 ± 0.43 B,a	16.45 ± 1.38 D,a	2.76 ± 0.36 E,a	<0.0001 ***
	15–30 cm	14.08 ± 1.45 C,b	23.66 ± 3.51 A,b	24.55 ± 0.84 B,b	11.12 ± 3.16 D,b	3.14 ± 0.65 E,a	
	30–45 cm	15.30 ± 1.54 B,b	20.17 ± 3.46 A,b	19.30 ± 2.74 A,c	9.02 ± 3.06 C,c	2.15 ± 0.45 E,b	
*p*-value	<0.0001 ***						
Mg^2+^ (meq/L)	0–15 cm	35.40 ± 2.73 B,a	60.63 ± 8.30 A,a	8.25 ± 0.43 C,a	5.77 ± 0.39 C,b	1.67 ± 0.25 D,b	<0.0001 ***
	15–30 cm	16.10 ± 0.89 B,b	33.74 ± 3.28 A,b	8.56 ± 0.36 D,a	13.51 ± 0.95 C,a	2.80 ± 0.24 E,a,b	
	30–45 cm	6.96 ± 0.53 C,c	11.49 ± 0.55 A,b	5.83 ± 0.60 D,b	10.34 ± 1.03 B,a	2.55 ± 0.32 E,a	
*p*-value	<0.0001 ***						
Na^+^ (meq/L)	0–15 cm	45.05 ± 6.52 B,a	134.81 ± 33.36 A,a	30.57 ± 0.71 B,C,a	5.12 ± 0.06 C,b	5.38 ± 0.61 C,a	<0.0001 ***
	15–30 cm	25.18 ± 4.89 B,b	44.34 ± 13.19 A,b	18.04 ± 1.09 B,C,b	15.53 ± 4.70 C,a	3.76 ± 0.60 D,b	
	30–45 cm	15.43 ± 2.41 B,b	27.34 ± 6.36 A,b	9.73 ± 2.83 C,c	13.49 ± 5.22 B,C,a	4.54 ± 0.18 c	
*p*-value	<0.0001 ***						
K^+^ (meq/L)	0–15 cm	14.63 ± 4.24 B,a	20.22 ± 4.97 A,a	2.16 ± 0.04 C,a	0.61 ± 0.11 D,c	0.51 ± 0.09 D,b	0.0001 ***
	15–30 cm	2.78 ± 0.47 B,b	1.42 ± 0.34B C,b	1.75 ± 0.08B C,b	9.95 ± 2.98 A,a	2.57 ± 0.08 B,a	
	30–45 cm	1.88 ± 0.49 B,c	1.52 ± 0.36 B,C,b,	1.25 ± 0.40 B,C,b	3.34 ± 2.36 A,b	0.52 ± 0.05 C,b	
*p*-value	<0.0001 ***						
Cl^−^ (meq/L)	0–15 cm	96.90 ± 13.08 B,a	237.60 ± 41.19 A,a	66.36 ± 1.27 B,C,a	13.04 ± 1.24 C,D,c	8.65 ± 0.78 D,a	<0.0001 ***
	15–30 cm	48.02 ± 4.62 B,b	73.30 ± 18.32 A,b	48.00 ± 1.76 B,C,b	41.60 ± 10.16 C,a	7.25 ± 1.42 D,b	
	30–45 cm	30.94 ± 3.40 B,c	51.00 ± 11.90 A,c	25.67 ± 8.35 B,C,c	32.58 ± 9.77 B,a,b	7.07 ± 0.78 C,b	
*p*-value	<0.0001 ***						
HCO_3_^−^ (meq/L)	0–15 cm	3.00 ± 0.137 A,a	3.58 ± 0.54 A,a	2.43 ± 0.21 A,a	1.13 ± 0.03 B,b	1.22 ± 0.03 B,b	0.2300 ns
	15–30 cm	1.51 ± 0.130Bb	2.36 ± 0.18Aa	0.9 ± 0.12Cb	1.38 + 0.15Bb	2.09 ± 0.27Aa	
	30–45 cm	1.14 ± 0.091 B,b	2.13 ± 0.19 A,a	1.09 ± 0.05 B,a	4.14 ± 2.55 A,a	1.47 ± 0.19 B,b	
*p*-value	0.3849						
SO_4_^2−^ (meq/L)	0–15 cm	15.04 ± 4.32 B,a	14.19 ± 3.03 B,C,b	3.92 ± 0.61 D,b	12.84 ± 0.16 C,a	71.80 ± 0.05 A,a	<0.0001 ***
	15–30 cm	6.36 ± 1.39 B,b	27.13 ± 1.14 A,a	3.05 ± 0.59 C,b	1.86 ± 0.43 D,b	0.96 ± 0.19 E,b	
	30–45 cm	10.97 ± 1.77 A,a,b	9.42 ± 2.52 A,c	8.14 ± 2.60 B,a	1.13 ± 0.56 C,b	0.60 ± 0.07 D,b	
*p*-value	0.0058 **						
OM (%)	0–15 cm	1.63 ± 0.19 A,a	1.78 ± 0.37 A,a	0.55 ± 0.02 B,b	0.81 ± 0.12 B,a	0.33 ± 0.04 B,b	<0.0001 ***
	15–30 cm	0.98 ± 0.17 A,b	0.87 ± 0.20 A,b	0.35 ± 0.02 C,a	0.45 ± 0.14 B,b	0.44 ± 0.12 B,a	
	30–45 cm	0.91 ± 0.33 A,b	0.54 ± 0.06 B,c	0.98 ± 0.29 A,a	0.39 ± 0.13 C,c	0.26 ± 0.03 D,c	
*p*-value	0.0003 ***						
CaCO_3_ (%)	0–15 cm	34.84 ± 2.10 A,b	18.02 ± 2.01 D,a	20.77 ± 0.77 C,b	32.58 ± 0.14 B,a	1.23 ± 0.09 E	<0.0001 ***
	15–30 cm	36.62 ± 0.94 A,b	16.76 ± 1.04 C,b	18.31 ± 0.55 B,c	4.08 ± 0.56 D,b	0.00 ± 0.00 E	
	30–45 cm	45.35 ± 1.68 A,a	17.01 ± 0.88 C,c	29.36 ± 2.17 B,a	4.03 ± 0.78 D,b	0.00 ± 0.00 E	
*p*-value	<0.0001 ***						
Clay (%)	0–15 cm	12.48 ± 065 C,a	16.80 ± 1.26 A,a	15.14 ± 1.64 B,a	12.67 ± 0.08 C,a	14.33 ± 1.03B,a	0.0023 **
	15–30 cm	10.84 ± 0.64 C,b	12.72 ± 0.41 B,b	7.79 ± 0.13 D,c	11.20 ± 0.53 B,b	13.58 ± 1.76 A,b	
	30–45 cm	10.60 ± 0.54 C,b	10.84 ± 0.83 C,c	11.20 ± 0.56 B,b	10.80 ± 0.83 C,c	12.32 ± 0.86 A,b	
*p*-value	<0.0001 ***						
Silt (%)	0–15 cm	17.28 ± 1.86 C,b	46.40 ± 2.15 A,b	6.88 ± 1.17 E,b	15.25 ± 1.04 D,c	36.70 ± 1.88 B,b	<0.0001 ***
	15–30 cm	22.48 ± 1.49 D,a	54.77 ± 1.46 A,a	2.04 ± 0.27 E,c	32.28 ± 1.37 C,a	41.30 ± 2.58 B,a	
	30–45 cm	22.68 ± 1.51 D,a	32.54 ± 1.75 A,b	12.10 ± 1.41 E,a	26.08 ± 2.56 C,b	29.61 ± 2.26 B,c	
*p*-value	<0.0001 ***						
Sand (%)	0–15 cm	70.24 ± 2.50 C,a	36.80 ± 1.634 E,b	78.58 ± 1.46 A,b	74.41 ± 2.52 B,a	48.68 ± 2.11 D,b	<0.0001 ***
	15–30 cm	66.68 ± 1.23 B,b	34.51 ± 0.99 E,b	90.17 ± 0.36 A,a	56.53 ± 1.83 C,c	45.12 ± 2.41 D,c	
	30–45 cm	66.72 ± 0.72 B,b	56.62 ± 1.49 D,a	76.70 ± 1.74 A,b	63.12 ± 2.72 C,b	58.07 ± 2.36 D,a	
*p*-value	<0.0001 ***						
MC (%)	0–15 cm	7.06 ± 0.55 B,a	28.83 ± 0.75 A,a	5.78 ± 0.44 B,a	4.97 ± 1.01 B,a	1.64 ± 0.25 C,c	<0.0001 ***
	15–30 cm	5.14 ± 0.62 B,b	19.40 ± 0.79 A,a	3.80 ± 0.81 C,c	4.79 ± 1.08 C,b	2.55 ± 0.52 C,b	
	30–45 cm	3.34 ± 0.40 B,c	23.88 ± 0.96 A,a	3.75 ± 0.80 B,b	4.60 ± 0.88 B,b	3.45 ± 0.52 B,c	
*p*-value	<0.0001 ***						

Different capital letters showed significant variation among regions at *p* < 0.05 (Duncan’s test), with df 4 for the region and 4 for soil layers, respectively. Different small letters revealed significant differences among soil layers (0–15 cm, 15–30 cm, and 30–45 cm). Capital letters indicate the significance of regions and small letters soil layers, EC: electrical conductivity, OM: organic matter, MC: moisture content, *p < 0.05, ** *p* < 0.01, *** *p* < 0.001, and “ns” for *p* > 0.05.

**Table 2:** Single-factor analysis of variance (ANOVA) showing the effect of different saline regions on plant functional traits and biomass allocation of *A. lagopoides* having a degree of freedom of the studied regions.

Functional Traits	Unit	SS	MS	*F* Value	*p* Value
Shoot length	cm	1901.38	475.34	40.56	<0.0001 ***
Shoot fresh weight	g	2221.49	555.37	5.04	0.0019 **
Shoot dry weight	g	1182.11	295.52	5.91	0.0006 ***
Number of tillers/plant	No.	2251.37	562.84	42.67	<0.0001 ***
Number of stolons/plant	No.	151.02	37.75	7.10	0.0002 ***
Average stolon length	cm	22701.03	5675.25	25.97	<0.0001 ***
Root length	cm	773.21	193.30	14.38	<0.0001 ***
Root fresh weight	g	92.62	23.15	6.25	0.0004 ***
Root dry weight	g	63.22	15.80	6.89	0.0002 ***
Root area	cm^2^	5335.64	1333.91	8.10	0.0001 ***
Leaf fresh weight	g	36182.34	9045.58	13.46	<0.0001 ***
Leaf dry weight	g	24768.00	6192.00	675.49	<0.0001 ***
Number of leaves/plant	No.	342601.02	85650.25	8.28	<0.0001 ***
Specific leaf area	cm^2^/g	0.05	0.01	7.53	0.0001 ***
Leaf dry matter content	g	39.38	9.84	6.63	0.0003 ***
Leaf thickness	µm	223.61	55.90	1.28	0.2902 NS
Number of spikes/plant	No.	12999.52	3249.88	6.07	0.0005 ***
Average. spike length	cm	2.10	0.52	3.35	0.0174 *
Spike fresh weight	g	0.03	0.01	14.42	<0.0001 ***
Spike dry weight	g	0.01	0.01	4.05	0.0068 **
Number of seeds/plant	No.	75 × 10^7^	1.87	48.56	<0.0001 ***

SS (Sum of Squares), MS (Mean Square), * *p* < 0.05, ** *p* < 0.01, *** *p* < 0.001, and “NS” for *p* > 0.05.

## Data Availability

Not applicable.

## Supplementary Materials

The following supporting information can be downloaded at: https://www.mdpi.com/article/10.3390/biology12040553/s1, Table S1: Geographical addresses of distinct patches of *A. lagopoides* populations of the studied regions of Saudi Arabia, along with yearly climatic data. Figure S1: Map of Saudi Arabia showing sampled regions of *Aeluropus lagopoides* populations; Figure S2: Pairwise geographical distance between the studied regions; Figure S3. Pearson’s Correlation heatmap between the top, middle, and bottom layer soil parameters and the different morphological and reproductive traits of *Aeluropus lagopoides* within different saline flat regions.
