# Supplementary material for: Phenotypic Plasticity Strategy of Aeluropus lagopoides Grass in Response to Heterogenous Saline Habitats
Source: Biology (Basel). 2023 Apr 5;12(4):553. doi: 10.3390/biology12040553 (PMC10135548; doi:10.3390/biology12040553)
Supplement: Supplementary file 1 [file biology-12-00553-s001.zip › biology-2271078-supplementary.pdf]

biology

Article

Phenotypic Plasticity Strategy of *Aeluropus lagopoides* Grass in Response to Heterogenous Saline Habitats

Abdulaziz M. Assaeed, Basharat A. Dar, Abdullah A. Al-Doss, Saud L. Al-Rowaily, Jahangir A. Malik and Ahmed M. Abd-ElGawad *

Plant Production Department, College of Food and Agriculture Sciences, King Saud University, Riyadh 11451, Saudi Arabia
* Correspondence: aibrahim2@ksu.edu.sa; Tel.: +966-5626-80864

Citation: Assaeed, A.M.; Dar, B.A.; Al-Doss, A.A.; Al-Rowaily, S.L.; Malik, J.A.; Abd-ElGawad, A.M. Phenotypic Plasticity Strategy of *Aeluropus lagopoides* Grass in Response to Heterogenous Saline Habitats. *Biology* **2023**, *12*, 553. https://doi.org/10.3390/biology12040553

Academic Editors: Daniel Puppe, Panayiotis Dimitrakopoulos, Baorong Lu and Caifu Jiang

Received: 23 February 2023
Revised: 28 March 2023
Accepted: 3 April 2023
Published: 5 April 2023

Copyright: © 2023 by the authors. Submitted for possible open access publication under the terms and conditions of the Creative Commons Attribution (CC BY) license (https://creativecommons.org/licenses/by/4.0/).

Supplementary Materials

Table S1. Geographical addresses of distinct patches of *A. lagopoides* populations of the studied regions of Saudi Arabia along with yearly climatic data.

Region	Location	Patch No.	Coordinates N	Coordinates E	Elevation m a.s.l	Yearly Min. Temp (°C)	Yearly Max. Temp (°C)	Yearly Humidty (%)	Yearly Rainfall (mm)
**Al-Jouf**	Inland saline flat region in Domat Aljandal	1	29°49.200	039°58.393	565	14.23	28.42	29.75	3.58
		2	29°49.270	039°58.457	563				
		3	29°49.850	039°58.931	558				
		4	29°49.085	039°58.149	519				
		5	29°49.169	039°57.494	525				
**Jizan**	Coastal saline flat region on the Southern Coastal Region	1	16°58.102	042°33.849	15	27.23	32.51	69.67	12.67
		2	16°58.122	042°33.707	9				
		3	16°58.137	042°33.667	6				
		4	16°58.144	042°34.082	4				
		5	16°58.114	042°34.016	4				
**Salwa**	Coastal saline flat region as lowland on the coast of the Arabian Gulf	1	24°45.392	050°45.225	-10	19.35	34.25	29.92	6.08
		2	25°43.664	050°08.274	-9				
		3	25°43.759	050°08.045	-8				
		4	24°45.071	050°45.348	-11				
		5	25°43.664	050°08.274	-9				
**Riyadh**	Inland saline flat region in wadi Hargan, Qareenah	1	25°03.995	046°10.795	833	18.50	32.70	22.75	5.50
		2	25°03.975	046°10.802	824				
		3	25°03.944	046°10.824	816				
		4	25°03.923	046°10.852	812				
		5	25°03.890	046°10.897	810				
**Al-Qassim**	Inland saline flat region of the Al-Aushazia location	1	26°03.295	44°08.168	590	17.31	32.22	24.33	7.75
		2	26°03.309	44°08.253	654				
		3	26°03.236	044°08.220	621				
		4	26°03.770	044°08.272	603				
		5	26°03.351	044°08.144	595				

Note:- N: Latitude; E: Longitude; m a.s.l : meter above sea level; Min: minimum Temp: Temperature; Max: Maximum and mm: millimeter. The data source for the yearly climate of the studied region had been taken from Climate data calculated by climate-data.org (Climate Saudi Arabia: Average Temperature, Weather by year & Weather for Saudi Arabia - Climate-Data.org).

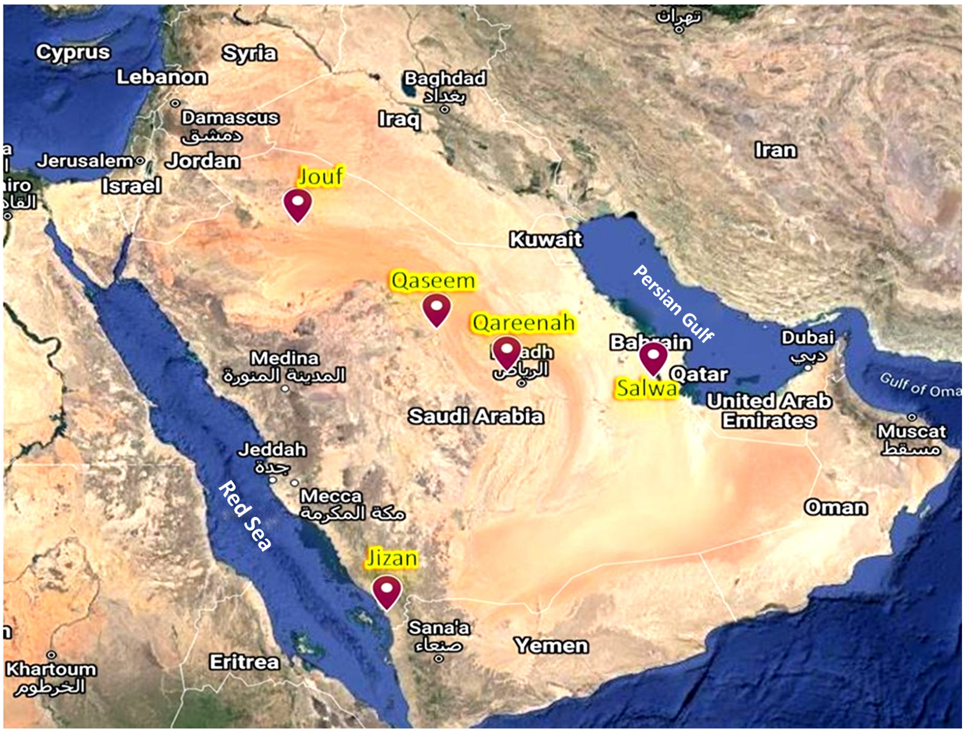

Figure S1. Map of Saudi Arabia showing sampled regions of *Aeluropus lagopoides* populations. The map is derived from Google Earth Pro, NVIDIA Corporation.

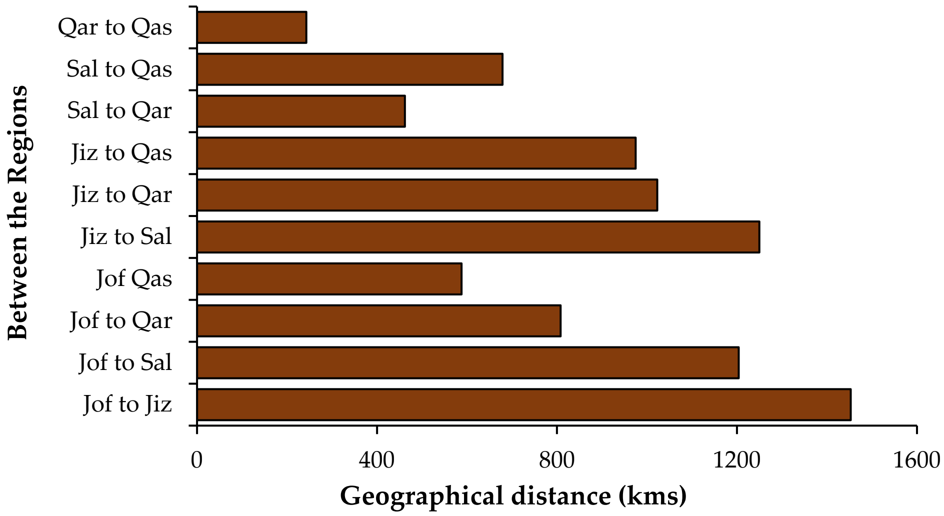

Figure S2. Pairwise geographical distance between the studied regions. Qar: Qareenah; Qas: Qaseem; Sal: Salwa; Jiz: Jizan and Jof: Jouf

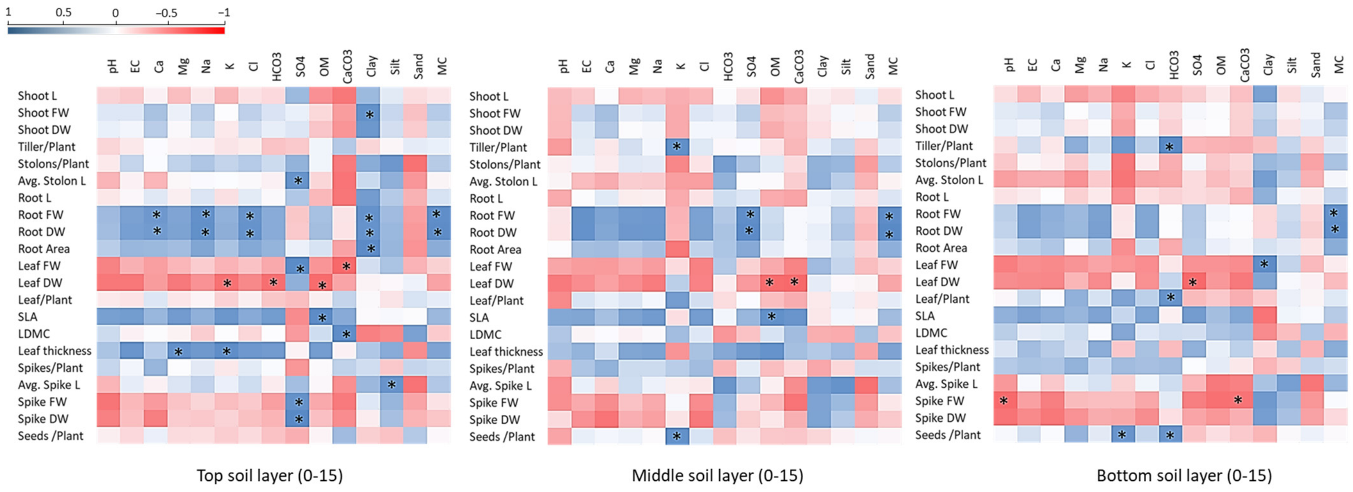

Figure S3. Pearson's Correlation heatmap between the soil parameters of the top, middle, and bottom layer and the different morphological and reproductive traits of *Aeluropus lagopoides* within different saline flat regions. * showed significant correlation values.
